# Yiqi Huoxue Tongluo recipe regulates NR4A1 to improve renal mitochondrial function in unilateral ureteral obstruction (UUO) rats

**DOI:** 10.1080/13880209.2022.2148168

**Published:** 2022-11-25

**Authors:** Zhen He, Mengjuan Zhang, Hepeng Xu, Wenping Zhou, Chang Xu, Zheng Wang, Ming He, Xiangting Wang

**Affiliations:** Hebei Key Laboratory of Integrative Medicine on Liver-Kidney Patterns, Institute of Integrative Medicine, College of Integrative Medicine, Hebei University of Chinese Medicine, Shijiazhuang, People’s Republic of China

**Keywords:** Mitochondra, renal injury, YHTR, apoptosis, TCA

## Abstract

**Context:**

Yiqi Huoxue Tongluo recipe (YHTR) is a traditional Chinese medicine for the treatment of chronic kidney disease, but its exact mechanism is not clear.

**Objectives:**

To monitor the potential improvement of renal mitochondrial function in unilateral ureteral obstruction (UUO) rats by regulating NR4A1 using the YHTR.

**Materials and methods:**

Wistar rats were randomly divided into four groups: sham, UUO (left ureteral ligation for 14 days), eplerenone (EPL) (UUO + EPL), and YHTR (UUO + YHTR). UUO rats were established and intragastrically administered EPL (100 mg/day/kg) or YHTR (11.7 g/day/kg) for 14 days. The expression of related proteins in kidneys was detected by immunohistochemistry, western blot, RT-PCR, and chemical colorimetric assay, respectively.

**Results:**

*In vivo*, YHTR treatment reduced the levels of BUN and Scr (by 17.9% and 23.5%) in UUO rats. Moreover, YHTR improved the renal mitochondrial function *via* increasing key enzymes of the tricarboxylic acid (TCA) cycle (*p* < 0.05) and activity of the mitochondrial complex (I–V) (by 30.8%, 29.1%, 19.7%, 35.9%, and 22.4%) in UUO rats. Compared with the UUO group, the expression of NR4A1 and Bcl-2 were significantly increased (*p* < 0.05), the expression of caspase-3 and caspase-9 were significantly decreased (*p* < 0.05) in the YHTR group. YHTR could upregulate key enzymes of the TCA cycle *via* promoting NR4A1 expression in HK2 cells, leading to inhibition of TGF-β1 induced cell apoptosis.

**Conclusions:**

YHTR significantly improved the development of CKD; this study may provide new ideas for the pathogenesis of CKD and new strategies for the development of new drugs against CKD.

## Introduction

Chronic kidney disease (CKD) is a common chronic disease characterized by structural damage and functional decline of the kidneys, which affects 697.5 million people worldwide and up to 132 million in China, and is a major clinical and public health problem (GBD Chronic Kidney Disease Collaboration 2020). Studying the pathogenesis of CKD is of great significance for finding effective treatments.

The mitochondrion is a small organelle exclusively of maternal inheritance and is directly involved in many essential cellular functions, including generating ATP through the tricarboxylic acid (TCA) cycle and mitochondrial electron transport chain (ETC)-mediated oxidative phosphorylation (OXPHOS) to meet the energy required for cell survival, promoting growth and metabolism (Wallace 1999; Newmeyer and Ferguson-Miller 2003). The kidney has the second highest mitochondrial content and oxygen consumption after the heart. In the kidney, proximal tubules require abundant mitochondria to provide sufficient energy for ATP, which required for tubular reabsorption and secretion, and are highly dependent on OXPHOS (Weinberg et al. 2000). Therefore, the kidneys are exquisitely dependent on mitochondria and are susceptible to mitochondrial damage (Che et al. 2014). Evidence indicates that mitochondrial dysfunction is not only an early event in kidney injury but also contributes to the development and progression of CKD (Sivitz and Yorek 2010; Sharma et al. 2013). Mitochondrial dysfunction leads to proteinuria increase (Su et al. 2013), NLRP3 inflammasome activation (Gong et al. 2016), uraemic toxin stagnation (Gewin et al. 2017), and transforming growth factor-β (TGF-β) expression (Casalena et al. 2012). Mitochondria are becoming a popular topic in CKD treatment, and the exploration of renal tubular mitochondrial function in CKD might provide an effective treatment strategy for CKD.

Nuclear receptor subfamily 4 group A member 1 (NR4A1) belongs to the nuclear receptor superfamily and regulates cellular functions in various forms, including cell proliferation, apoptosis, differentiation, development, immunity, and metabolism, and plays an important role in energy metabolism in particularly (Safe et al. 2016). Recent studies reported that NR4A1 can ameliorate age-related renal tubulointerstitial fibrosis by suppressing the TGF-β/Smads signalling pathway in myofibroblasts, suggesting that NR4A1 is a potential therapeutic target for age-related kidney diseases including CKD (Ma et al. 2022). NR4A1 is closely related to mitochondrial function. NR4A1 promotes glucose uptake, glycolysis, phosphoglycerol shuttle, and glycogenolysis (Pei et al. 2006; Chao et al. 2007). Whether NR4A1 prevents CKD progression by improving mitochondrial function requires further study.

Yiqi Huoxue Tongluo recipe (YHTR) is an herbal decoction consisting of eight herbs, including *Astragali Radix, Cicadae Periostracum, Pheretima, Bombyx Batryticatus, Zaocys, Salviae miltiorrhizae Radix et Rhizoma, Chuanxiong Rhizoma* and *Testudinis Carapax et Plastrum.* According to traditional Chinese medicine theory, YHTR has the effect of benefiting Qi, invigorating blood and promoting blood circulation. YHTR has been used clinically as a basic formulation for the treatment of patients with CKD (Ding et al. 2011; Hao et al. 2019). However, the exact mechanism is unclear and requires further study. However, its underlying pharmacological mechanism remains unclear.

In this study, we will investigate the effect of YHTR on kidney of unilateral ureteral obstruction (UUO) rat model, to determine the NR4A1 role in association with the pro-mitochondrial function which will further deepen the understanding of the pathophysiology of CKD and provide new ideas for the treatment of CKD using the YHTR.

## Materials and methods

### Drug preparation

The YHTR was composed of Huangqi (*Astragali Radix*) 30 g, Chantui (*Cicadae Periostracum*) 10 g, Dilong (*Pheretima*) 12 g, Jiangcan (*Bombyx Batryticatus*) 10 g, Wushaoshe (*Zaocys dhumnades*) 10 g, Danshen (*Salviae miltiorrhizae Radix et Rhizoma*) 15 g, Chuanxiong (*Chuanxiong Rhizoma*) 10 g, and Guiban (*Testudinis Carapax et Plastrum*) 15 g (Table 1). Those herbal granules were purchased from Shineway Pharmaceutical Co., Ltd. (Shijiazhuang, China). The voucher specimens, identified by Professor Long Guo, have been deposited at Hebei University of Chinese Medicine, Shijiazhuang, China.

**Table 1.** The composition of YHTR.

Latin name	Species	Family	Batch number	Herb dose (g)	Ratio (%)
Astragali Radix	*Astragalus* L.	Fabaceae	21071121	30	26.8
Cicadae Periostracum	*Cryptotympana pustulata* Fabricius	Cicadidae	21021881	10	8.9
Pheretima	*Pheretima aspergillum*	Megascolecidae	21012421	12	10.7
Bombyx Batryticatus	*Bombyx mori* L.	Bombycidae	21041461	10	8.9
Zaocys dhumnades	*Zaocys*	Colubridae	20081171	10	8.9
Salviae miltiorrhizae Radix et Rhizoma	*Salvia miltiorrhiza* Bge.	Labiatae	21070231	15	13.4
Chuanxiong Rhizoma	*Ligusticum chuanxiong* Hort	Apiaceae	21020621	10	8.9
Testudinis Carapax et Plastrum	*Chinemys reevesi*	Testudinidae	19120961	15	13.4

### Analysis of YHTR

A certain amount of the sample was taken, slowly dissolved at 4 °C, and the metabolites were extracted from the sample by the extraction method, based on methanol acetonitrile solution, and a certain volume of supernatant was taken after protein precipitation, ready for the machine. Quality control samples are inserted in the sample queue to monitor and evaluate the stability of the system and the reliability of the experimental data. Chromatographic separation was performed, and with the mobile phase, each metabolite was eluted from the column in turn. It is then fed into the Q-Exactive HF-X high-resolution liquid-mass spectrometer and detected in both positive and negative ion modes of the ESI ion source, resulting in two sets of positive and negative ion data. XCMS software was used for the data pre-processing process. The final output is a qualitative and quantitative data sheet of metabolites.

### Animal studies

Animal housing and procedures used in this study were approved by the local Animal Care and Use Committee of Hebei University of Chinese Medicine (no. DWLL2020015). Wistar rats (male, 200 ± 10 g) were purchased from Beijing Vital River Laboratory Animal Technology Co., Ltd. (SCXK 2016-0011, *n* = 32), which were randomly assigned into four groups: sham group, UUO group, eplerenone (EPL) group, and YHTR group. The rats in the UUO, EPL, and YHTR groups underwent ligation surgery of the left lateral ureter. After ligating the upper 1/3 and middle 1/3 of the ureter, respectively, the ureter between the two ligated ends was cut and the skin was sutured layer by layer (Truong et al. 2011). In sham group, the left ureter was being exposed but not ligated. For the eplerenone group, the rats were given oral administration of mixed eplerenone on a diet (100 mg/kg/day) after UUO. The YHTR group was intragastrically administrated with 11.7 g/kg YHTR every day (Zhang et al. 2020). In contrast, rats in the UUO and sham groups were administrated intragastrically with the same volume of normal saline. All rat kidneys were obtained on day 14 after UUO surgery.

### Cell culture and treatments

HK2 cells were cultured with Roswell Park Memorial Institute-1640 (RPMI-1640) Medium containing 10% serum, 1% penicillin and streptomycin at 37 °C. After the density of HK2 cells reached 80%, the cells were carried out cell experiments.

Preparation of serum containing YHTR: Male wistar rats (*n* = 10, 6–7 weeks of age) were given intragastric 11.7 g/day/kg of THTR twice a day for 4 consecutive days and blood was collected from the femoral arteries bilaterally 1 h after the last intragastric administration. After standing at room temperature for 2 h, the supernatant was collected by centrifuged at 3000 rpm for 10 min. THTR-medicated serum were obtained by inactivating complement in a water bath at 56 °C for 30 min, filtering and sterilizing with a 0.22 μm sterile filter, which were stored the serum at −80 °C for later use.

### Adenovirus expression vector

Adenoviruses encoding NR4A1 (Ad-NR4A1) and control (Ad) were constructed by Shanghai Hanheng Biotechnology company.

### Renal function analysis

A serum sample from all rats was used to test renal function by using blood urea nitrogen (BUN) and serum creatinine (Scr) screening kits from Beckman Coulter (No. AUZ3562, No. AUZ3611).

### Histological analysis and immunohistochemistry

The rat kidneys were fixed in 4% paraformaldehyde, dehydrated with alcohol, and embedded in paraffin. Paraffin blocks were cut into 3 μm sections for haematoxylin and eosin (H&E) and Masson staining. After fixation and paraffin embedding, 3 μm sections were dewaxed in xylene for 30 min, dehydrated in an alcohol gradient, 3% hydrogen peroxide was added for 10 min to block endogenous peroxidase, followed by antigen repair by heating in 10 mM of citrate buffer. Next, 10% normal goat serum was added to the sections for 10 min at 37 °C to block non-specific antibody binding. Then, sections were incubated with the citrate synthase (CS) (1:250, Abcam, No. GR246387-12), succinate dehydrogenase B (SDHB) (1:200, Proteintech, No. 10620-1-AP), NR4A1 (1:100, Immunoway, No. YT3213), Bcl-2 (1:200, Proteintech, No. 26593-1-AP), and caspase-3 (1:300, Proteintech, No. 66470-2-1 g), caspase-9 (1:50, Proteintech, No. 10380-1-AP) primary antibodies overnight at 4 °C. After washing with PBS, sections were incubated with enzyme-labeled goat anti-mouse/rabbit IgG polymer secondary antibody and horseradish peroxidase-conjugated streptavidin. Labels were visualized with diaminobenzidine (DAB) to produce a brown colour as a positive expression, and sections were counterstained with haematoxylin. Semi-quantitative analysis was also performed using Image J.

### Western blot analysis

The obstructed kidney tissues were homogenized for protein extraction. The proteins were separated by electrophoresis on 10% SDS-PAGE gels and then transferred to polyvinylidene difluoride membranes. Immunoblotting was performed with primary antibodies against CS (1:6000), SDHB (1:5000), caspase-9 (1:600), caspase-3 (1:1000), Bcl-2 (1:1500), NR4A1 (1:1000), GAPDH (1:2000, Servicebio, No. AC21030538), and β-actin (1:8000, Proteintech, No. 66009-1-lg) overnight. Next day, after the membranes incubated with secondary antibodies (room temperature, 1 h, 1:15,000), antibody-antigen complexes were visualized using a Chemiluminescence Plus Western immunoblot analysis kit (Millipore, USA).

### Quantitative real-time reverse transcription polymerase chain reaction (qRT-PCR)

Total RNA was isolated using the EZNA Total RNA Kit II (Omega, No. R6934-01) following the protocol recommended by the manufacturer. RT First Strand cDNA Synthesis Kit (Servicebio, No. LT203701) was used for reverse transcription of RNA; polymerase chain reaction was performed using the ABI 7,500 FAST System (Thermo, USA) with SYBR Green qPCR Master Mix (Servicebio, No. MPC2011004). The target genes were NR4A1(forward, 5′-TTGGAAAGGAAGATGCCGG-3′, reverse, 5′-TGTCTATCCAGTCACCAAAGCC-3′), CS (forward, 5′-TAAGCTGGACTGGTCCCACA-3′, reverse, 5′-TGGTTTGCTAGTCCATGCAGA-3′), GAPDH (forward, 5′-CTGGAGAAACCTGCCAAGTATG-3′, reverse, 5′-GGTGGAAGAATGGGAGTTGCT-3′). All primers were supplied by Servicebio Biology Science and Technology Co., Ltd. GAPDH was used as a housekeeping transcript for normalization, and relative real-time PCR results were calculated using the 2^–ΔΔCT^ method.

### Citrate synthase and succinate dehydrogenase assay

The protein supernatants from approximately 0.1 g of kidney tissue were collected, and used to detect the activity levels of CS (Njjcbio, No. A108-1-2) and SDH (Njjcbio, No. A022-1-1) according to the Nanjing Jiancheng Company kit, which reflect the level of mitochondrial function in kidney.

### Electron transport chain complexes I–V assay

The activity of complex I, II, III, IV, and V in the mitochondrial ETC was detected by chemical colorimetry (Beijing Solaibao Biotechnology Co, Beijing, China, No. 20210329, No. 20210330, No. 20210319, No. 20210316, No. 20210317) following the manufacturer’s instructions.

### Statistical analysis

All data were expressed as mean ± standard error of the mean (SEM), which were analyzed using SPSS version 23.0. The statistical comparisons between groups were determined with one-way analysis of variance followed by SNK (Student–Newman–Keuls) q-test when data were normally distributed and had equal variance. *p*-Values < 0.05 were considered statistically significant.

## Results

### Identification of main compositions of YHTR

High performance liquid chromatography analysis was performed to identify cryptotanshinone, ligustilide, ligustrazine, calycosin-7-glucoside, danshensu, and calycosin from YHTR. Our results indicate that these six compounds are important components of YHTR and scored the highest (Table 2).

**Table 2.** Components identified in YHTR.

rt (min)	*m*/*z*	Adduct	Formula	Source	Identification	Score	CASV
9.371	297.14676	[M + H]+	C_19_H_20_O_3_	*Salviae miltiorrhizae Radix et Rhizoma*	Cryptotanshinone	0.878	35825-57-1
8.029	191.10555	[M + H]+	C_12_H_14_O_2_	*Chuanxiong Rhizoma*	Ligustilide	0.859	4431-01-0
2.123	137.10587	[M + H]+	C_8_H_12_N_2_	*Chuanxiong Rhizoma*	Ligustrazine	0.747	1124-11-4
4.76	447.12918	[M + H]+	C_22_H_22_O_10_	*Astragali Radix*	Calycosin-7-glucoside	0.92	20633-67-4
5.059	197.04385	[M–H]–	C_9_H_10_O_5_	*Salviae miltiorrhizae Radix et Rhizoma*	Danshensu	0.89	831-61-8
6.205	283.06326	[M–H]–	C_16_H_12_O_5_	*Astragali Radix*	Calycosin	0.901	632-85-9

### YHTR improves UUO-injured renal function

**Figure 1.**
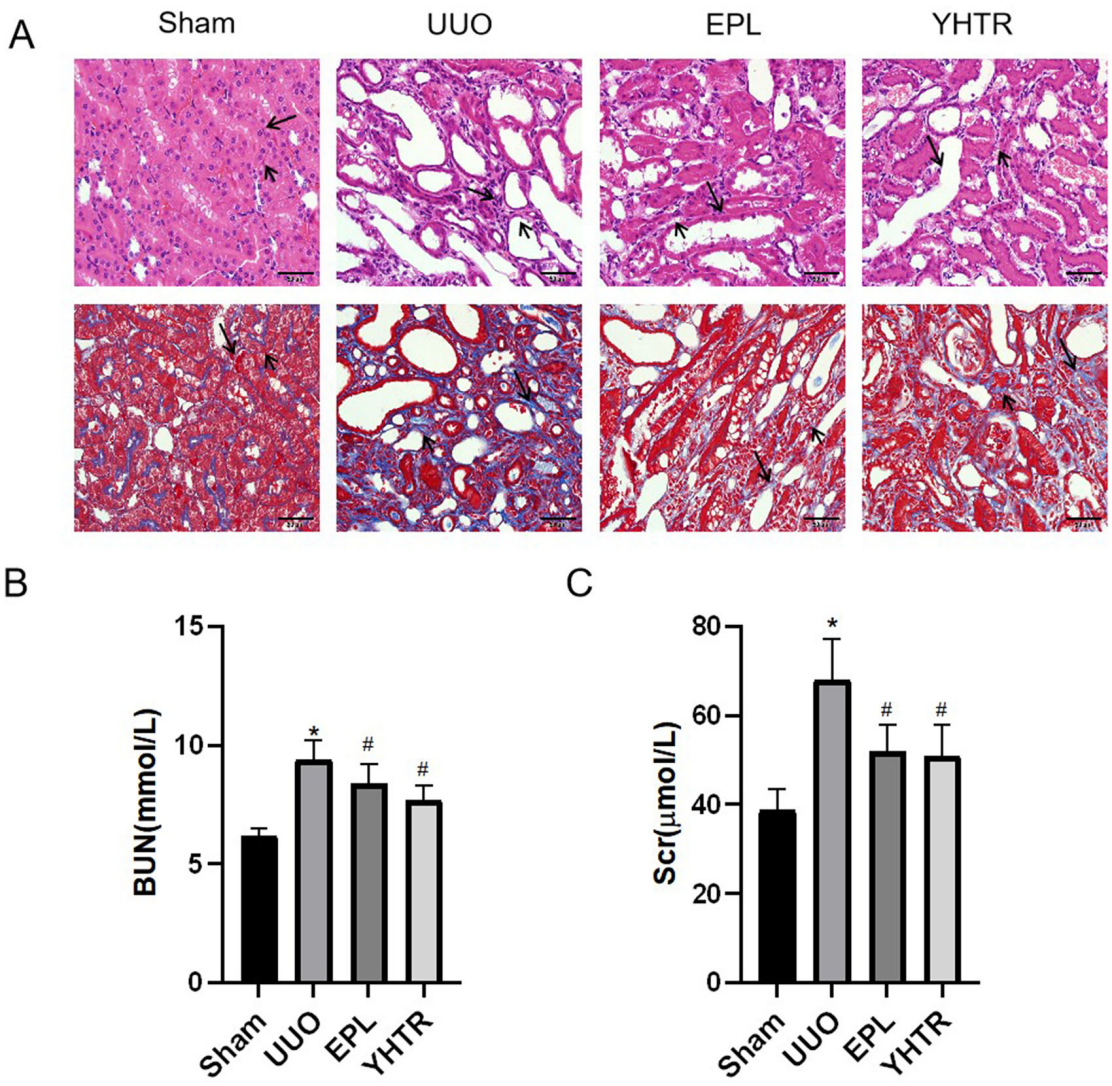
YHTR ameliorates pathological renal damage in rats after 14 days of UUO. (A) Representative haematoxylin–eosin (HE) and Masson stained kidney sections of each group of rats. Magnification, ×200, scale bar = 50 μm. (B) and (C) The levels of BUN and Scr in serum of each group of rats. Data are presented as the means ± SEM. *p < 0.05 vs. sham group, *^#^p* < 0.05 vs. UUO group.

We established the UUO rat model, which can lead to progressive CKD, and we evaluated the renal histomorphology by HE and Masson staining. As shown in Figure 1A, the renal tubular morphology in sham group was structurally normal with well-aligned tubular epithelial cells and a small amount of collagen deposition. There was a severe structural damage with a reduced number of renal tubules, tubular atrophy, and dilated lumen in UUO group. The renal tubular morphology in YHTR and EPL group were close to sham group. Compared with sham group, the levels of BUN and Scr in serum were significantly increased in UUO group, and decreased in YHTR and EPL group (Figure 1(B,C)). These results suggest that YHTR has the effect of improving kidney function.

### YHTR improves NR4A1 expression in the kidney of UUO rats

**Figure 2.**
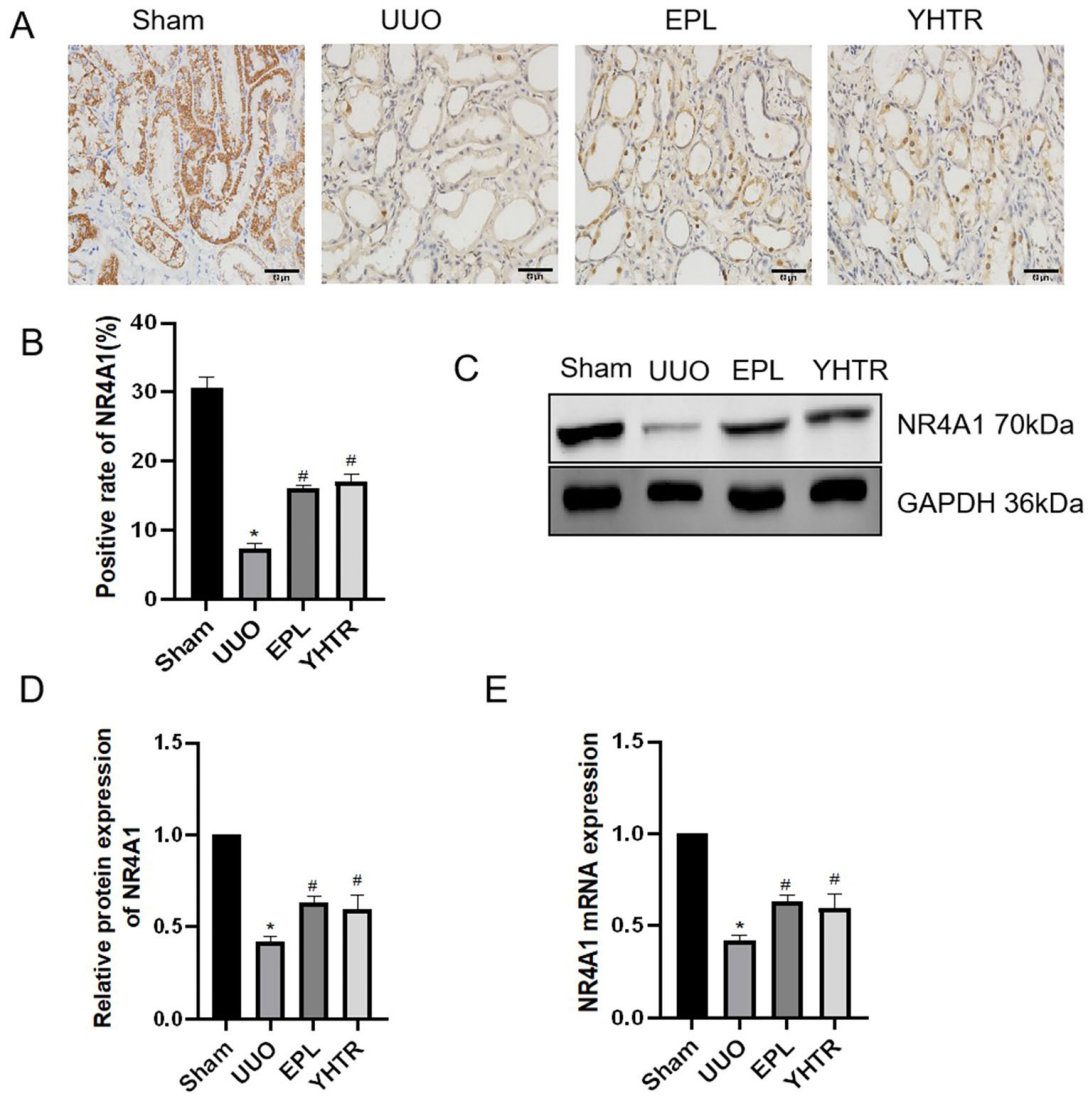
YHTR improves NR4A1 expression in the kidney of UUO rats. (A) Immunohistochemical (IHC) staining of NR4A1 in the kidney of each group of rats. Magnification, ×200, scale bar = 50 μm. (B) The mean intensity of IHC as shown in panel A. Data are presented as the means ± SEM. *p < 0.05 vs. sham group, *^#^p* < 0.05 vs. UUO group. (C, D) NR4A1 protein level was detected by western blot analysis. Data are presented as the means ± SEM. *p < 0.05 vs. sham group, *^#^p* < 0.05 vs. UUO group. (E) Analysis of NR4A1 mRNA expression detected by qRT-PCR. Data are presented as the means ± SEM. *p < 0.05 vs. sham group, *^#^p* < 0.05 vs. UUO group.

Subsequently, we investigated NR4A1 expression in the kidney of UUO rats. Immunohistochemistry assay showed that compared with sham group, the proportion of NR4A1-positive regions was significantly reduced in UUO group and increased significantly in YHTR and EPL group (Figure 2(A,B)). Western blot analysis was performed and showed that NR4A1 protein expression was inhibited in UUO group and significantly upregulated by both YHTR and eplerenone treatment (Figure 2(C,D)). The results of RT-qPCR analysis were similar to the results of western blot analysis (Figure 2(E)). These results suggest that NR4A1 expression of kidney tissue was significantly decreased in UUO rats, and YHTR and eplerenone could attenuate its decrease in UUO rats.

### YHTR upregulates the renal mitochondrial function in UUO rats

**Figure 3.**
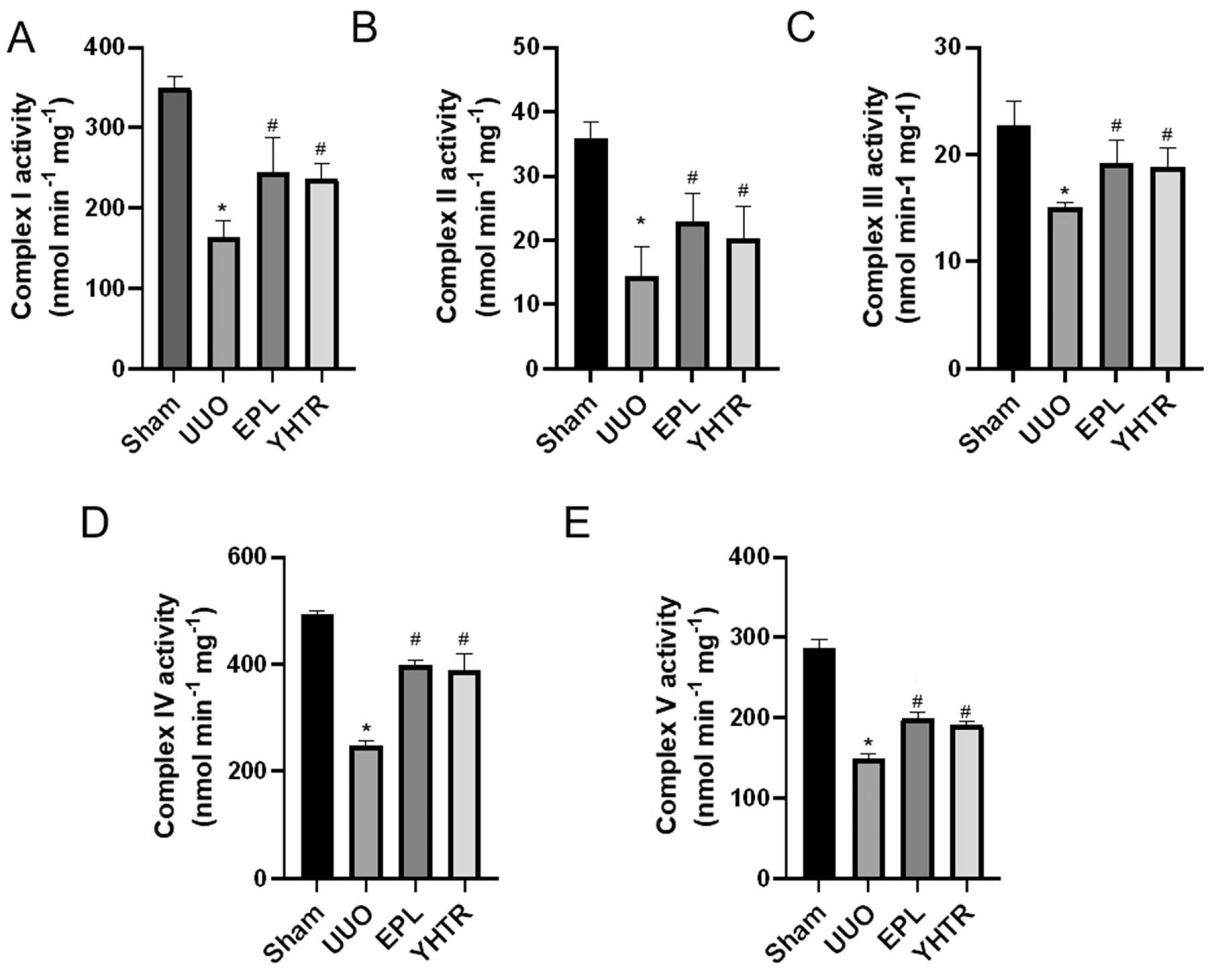
YHTR upregulates the renal mitochondrial ETC complex activity in UUO rats. (A) Mitochondrial ETC complex I activity. (B) Mitochondrial ETC complex II activity. (C) Mitochondrial ETC complex III activity. (D) Mitochondrial ETC complex IV activity. (E) Mitochondrial ETC complex V activity. Data are presented as the means ± SEM. *p < 0.05 vs. sham group; *^#^p* < 0.05 vs. UUO group.

The kidney is the second most oxygen-consuming organ in the body at rest. We have analyzed the expression of key enzymes of the mitochondrial TCA cycle and ETC in UUO rats in this study. Complex I, II, III, IV, and V activities were significantly lower in UUO group than that in sham group and significantly higher in YHTR and EPL group than that in UUO group, suggesting that UUO inhibited ATP formation and YHTR and eplerenone reversed the process (Figure 3(A–E)).

**Figure 4.**
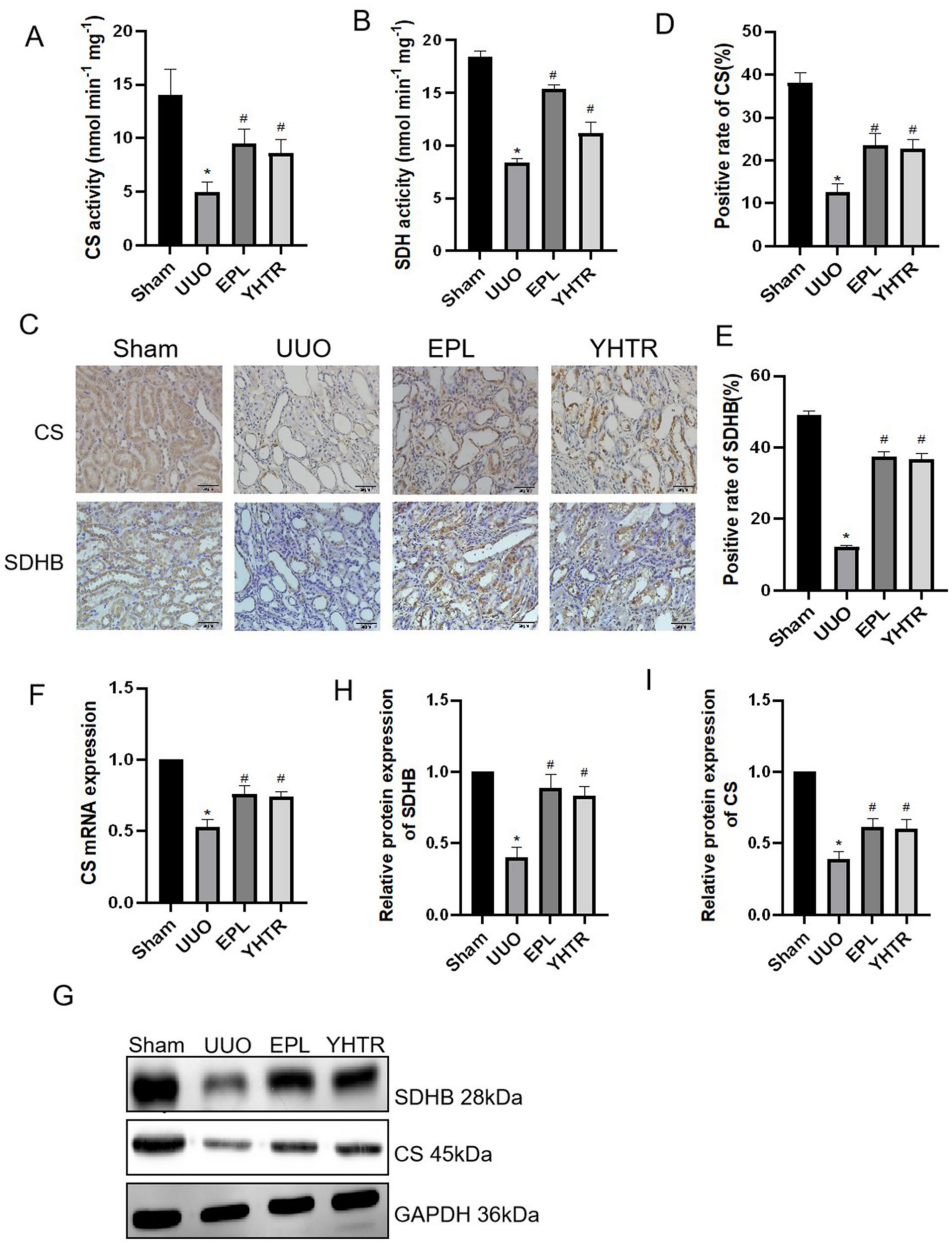
YHTR improved the renal mitochondrial function in rats 14 days after UUO. (A, B) The activities of CS and SDH in the kidney were measured by chemical colorimetric assay. Data are presented as the means ± SEM. *p < 0.05 vs. sham group, ^#^*p* < 0.05 vs. UUO group. (C–E) IHC staining of CS and SDHB in the kidney of each group of rats. Data are presented as the means ± SEM. *p < 0.05 vs. sham group; *^#^p* < 0.05 vs. UUO group. (F) CS mRNA expression in the kidneys detected by qRT-PCR analysis. Data are presented as the means ± SEM. *p < 0.05 vs. sham group, *^#^p* < 0.05 vs. UUO group. (G–I) Protein expression of CS and SDHB were detected by western blot analysis in the kidney of each group of rats. Data are presented as the means ± SEM. *p < 0.05 vs. sham group, *^#^p* < 0.05 vs. UUO group.

TCA produces ATP and CS, SDHB are key enzymes in this process. Chemical colorimetric analysis was performed and showed that the activities of CS, SDH were significantly reduced in the kidneys of rats after UUO compared with sham group, and the activities of these enzymes were recovered by YHTR and eplerenone treatment (Figure 4(A,B)). qRT-PCR analysis, immunohistochemical staining and western blot analysis were performed, similar results were obtained in the kidney, showing that CS and SDHB were markedly decreased in the kidney after UUO treatment and YHTR and eplerenone increased these gene expressions, respectively (Figure 4(C–I)). These results suggest that YHTR can improve renal mitochondrial function after UUO *via* regulating key enzymes of the mitochondrial TCA cycle and ETC.

### YHTR inhibits UUO-induced apoptosis

**Figure 5.**
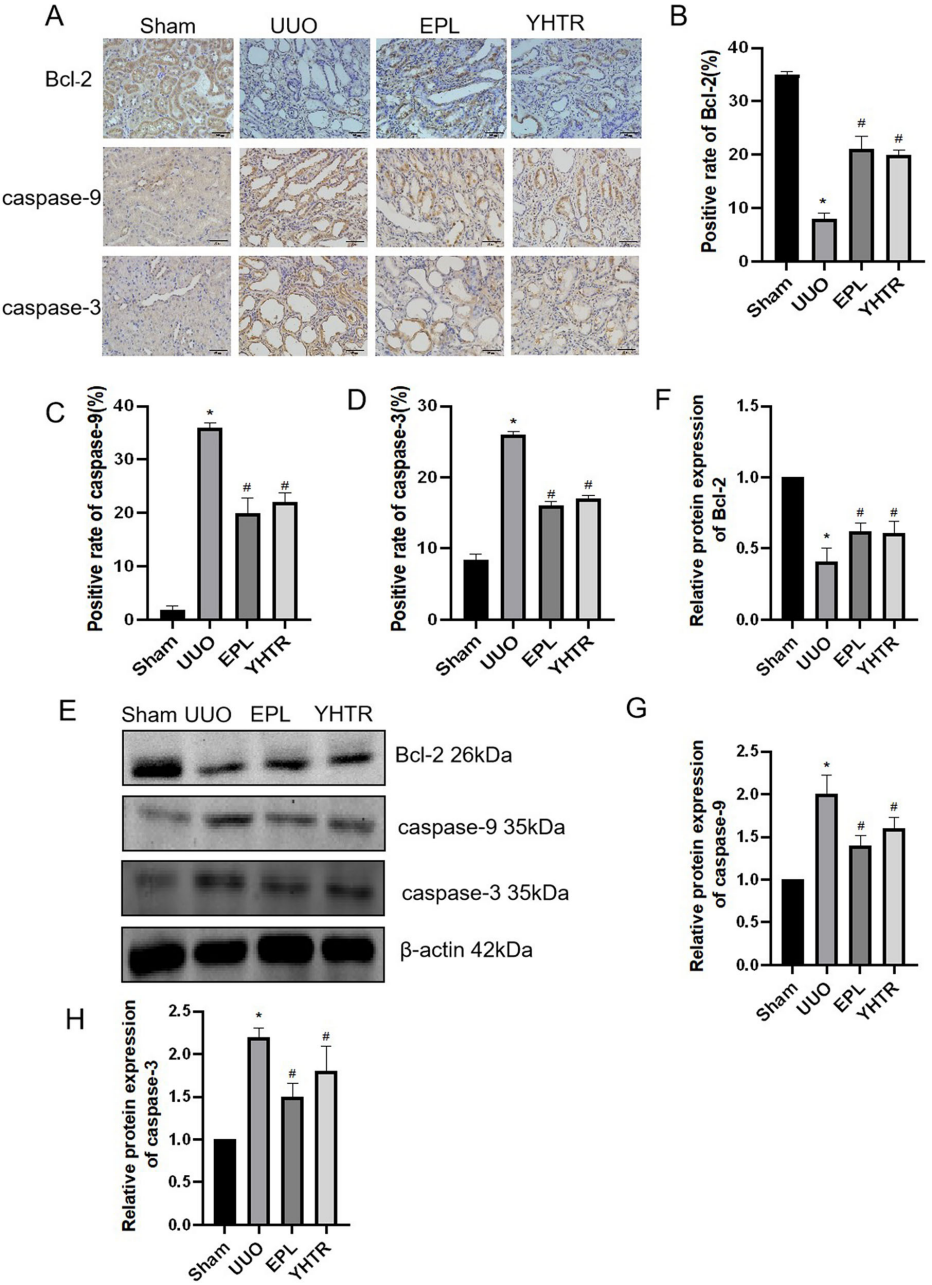
YHTR inhibited apoptosis in renal tissues of UUO rats. (A–D) IHC staining of Bcl-2, caspase-9, and caspase-3 in the kidney of each group of rats. Data are presented as the means ± SEM. *p < 0.05 vs. sham group, *^#^p* < 0.05 vs. UUO group. (E–H) Protein expression of Bcl-2, caspase-9, and caspase-3 in the kidney of each group of rats were analyzed by western blot analysis. Data are presented as the means ± SEM. *p < 0.05 vs. sham group, *^#^p* < 0.05 vs. UUO group.

We performed immunohistochemical analysis to detect apoptosis and found that caspase-3 and caspase-9 protein expression were up-regulated and anti-apoptotic factor Bcl-2 protein expression was down-regulated in kidney of UUO rats compared with sham group. However, the treatment with YHTR and eplerenone significantly reduced caspase-3 and caspase-9 protein expression and upregulated Bcl-2 protein expression (Figure 5(A–D)). Similar results were also observed by using western blot for apoptosis-related proteins in these kidneys. These data suggest that YHTR and eplerenone can inhibit UUO-induced apoptosis (Figure 5(E–H)).

### YHTR improves the renal mitochondrial function in HK2 cells by regulating NR4A1

**Figure 6.**
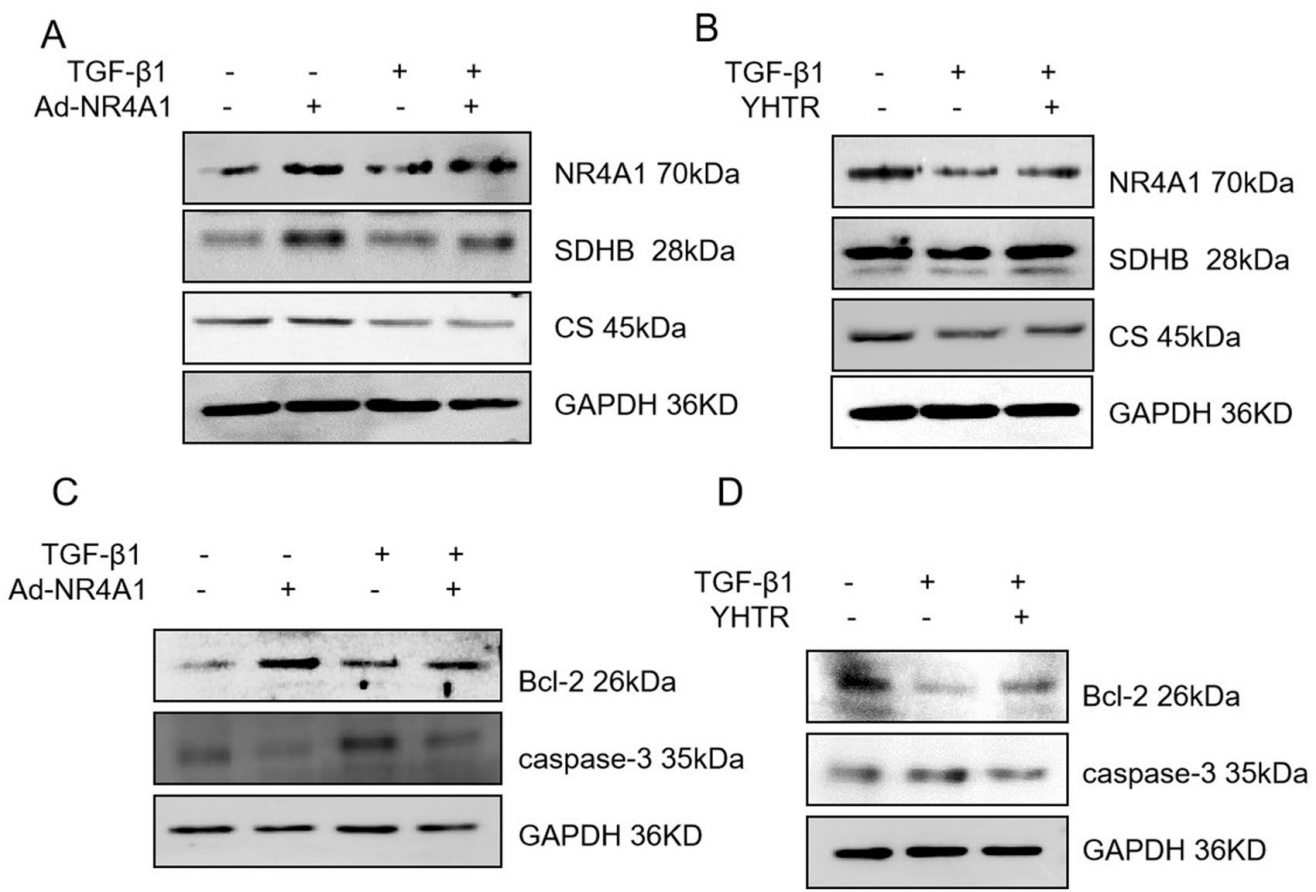
YHTR improves the renal mitochondrial function in HK2 cells by regulating NR4A1. (A) HK2 cells were infected with adenovirus vectors with NR4A1 24 h, or/and treated with TGF-β1 another 12 h. Western blot analysis was performed for NR4A1, CS and SDHB protein expression. (B) HK2 cells were incubated in medium containing YHTR serum for 12 h, then treated with TGF-β1 or vehicle for 12 h. Western blot analysis was performed for NR4A1, CS and SDHB protein expression. (C) After HK2 cells were infected with Ad-NR4A1 24 h and stimulated with TGF-β1 for 12 h, the protein expression of Bcl-2 and caspase-3 were detected by western blot analysis. (D) After HK2 cells were treated with serum containing YHTR 12 h and stimulated with TGF-β1 for 12 h, the protein expression of Bcl-2 and caspase-3 were detected by western blot analysis.

HK2 cells were infected with adenovirus vectors with NR4A1 and treated with TGF-β1. Western blot analysis was performed for CS and SDHB expression in HK2 cells. As shown in Figure 6A, NR4A1 overexpression increased CS and SDHB expression, while TGF-β1 decreased CS and SDHB expression in HK2 cells. HK2 cells were treated with TGF-β1 for 12 h following YHTR pre-treatment. Western blot analysis showed that the expression of CS and SDHB were inhibited by TGF-β1, and promoted by YHTR incubation (Figure 6B), suggesting that NR4A1 is essential for YHTR improving renal mitochondrial function in HK2 cells. Also, we detected the caspase-3 and Bcl-2 in HK2 cells by western blot analysis. The results showed that Bcl-2 protein expression was increased and caspase-3 protein expression was decreased in Ad-NR4A1 group. TGF-β1 reduced Bcl-2 protein expression and promoted caspase-3 protein expression in HK2 cells (Figure 6C). YHTR preincubation could upregulate Bcl-2 protein expression and downregulate caspase-3 protein expression (Figure 6D). These results indicated that YHTR could significantly trigger NR4A1 expression, which improved renal mitochondrial function in HK2 cells by promoting expression of CS and SDHB, and leading to inhibition of TGF-β1-induced cell apoptosis.

## Discussion

**Figure 7.**
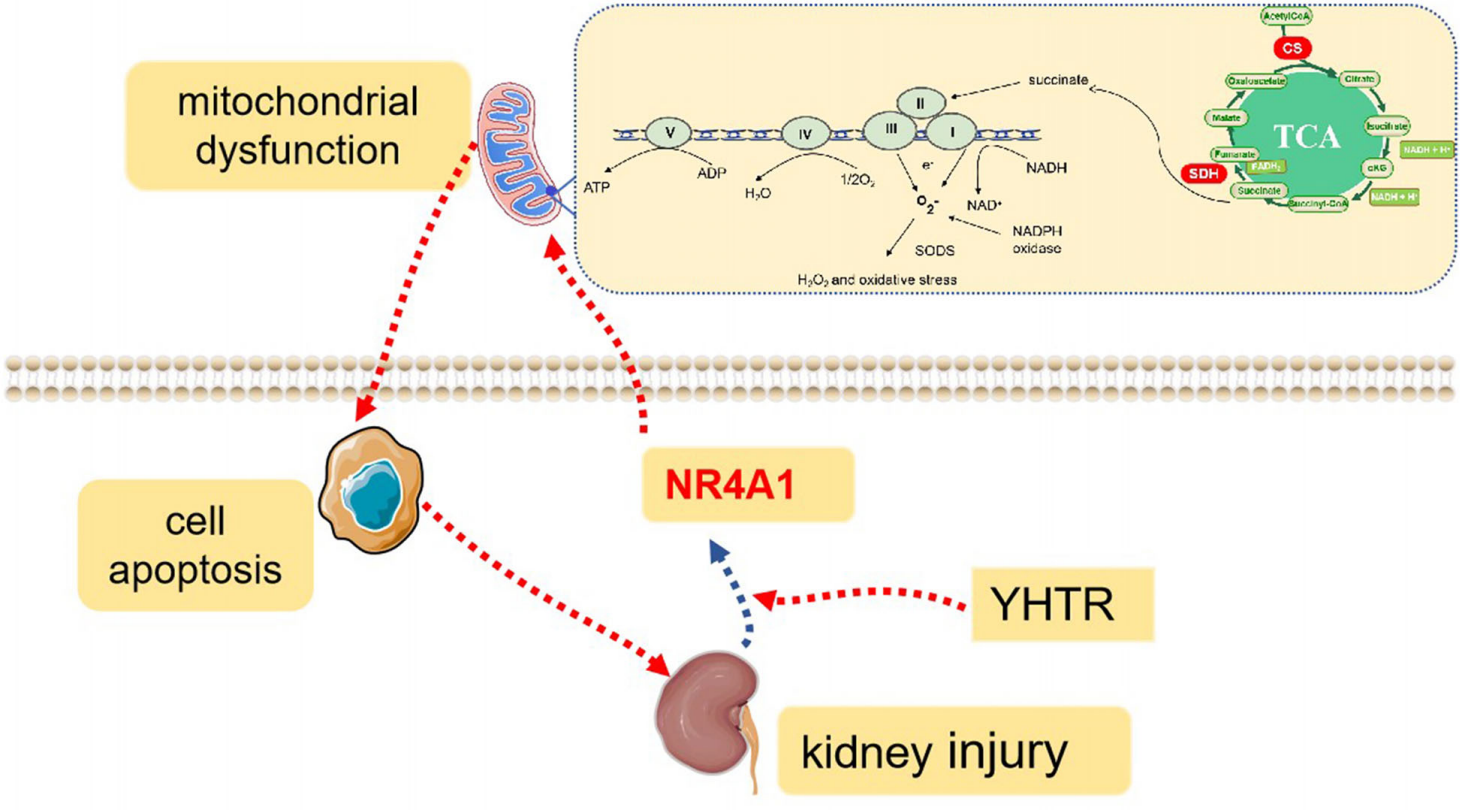
Proposed model for the mechanism of YHTR ameliorated pathological renal injury *via* promoting NR4A1 expression. In response to renal injury, TGF-β1 inhibited NR4A1 expression, caused mitochondrial dysfunction and led to cell apoptosis. YHTR could improve mitochondrial function and inhibited apoptosis through upregulating NR4A1 expression, finally ameliorated the development and progression of CKD.

Obstructive nephropathy is a common clinical disease (Xu et al. 2014). UUO is a widely used model for the study of obstructive nephropathy, which causes the development of CKD (Chevalier et al. 2010). The present study successfully replicated the characteristics of CKD in UUO rat model, as evidenced by decreased renal function and impaired renal structure. Our data showed that YHTR improved renal function and ameliorated pathological renal injury *via* promoting NR4A1 expression, which regulating mitochondrial energy metabolism and mitochondrial function in UUO rats (Figure 7).

Our previous studies have shown that YHTR can enhance the immunity of the body, inhibit oxidative stress and apoptosis, reduce extracellular matrix deposition, improve renal function and delay the process of interstitial fibrosis, which is important for protecting kidney function (Pan et al. 2008; Huang D 2016). In the present experiment we found that YHTR could improve renal function and pathological renal injury caused by UUO (Figure 1). Studies also suggested that eplerenone has antioxidant effects and might have protective effects against and delay the development of CKD (Bayorh et al. 2011). Also, eplerenone could ameliorate CKD progression (Figure 1).

The nuclear receptor superfamily includes at least 48 transcription factors that regulate a variety of cellular and metabolic functions in different biological processes (Chawla et al. 2001). The NR4A family is an orphan nuclear receptor and consists of three members: NR4A1 (NUR77), NR4A2 (NURR 1), and NR4A3 (NOR-1) (Nuclear Receptors Nomenclature Committee 1999). For these receptors, the endogenous ligands are not recognized (Kliewer et al. 1999). Therefore, the regulatory activity of the NR4A1 is ligand-independent, and the function of NR4A1 is receptor-dependent for expression and post-translational modifications (Wang et al. 2003). In Nr4a1–/– mice, the severity of tubular atrophy, tubular casts, and interstitial fibrosis were significantly increased, accompanied by massive infiltration of immune cells, mainly macrophages, T cells, and B cells (Zhang et al. 2018). In our study, we found that the expression of NR4A1 in kidney of UUO rats was increased and inhibited by YHTR and eplerenone (Figure 2).

Mitochondria are primarily responsible for ATP production *via* OXPHOS in the inner mitochondrial membrane. A significant increase in reactive oxygen species (ROS) production and abnormal respiratory chain complex expression were found in peripheral blood mononuclear cells of CKD patients, suggesting that mitochondrial dysfunction is closely related to CKD (Small et al. 2012). Response to kidney damage, mitochondria can disrupt ATP synthesis and activate the cell death pathway by producing excess ROS and releasing pro-death proteins that rapidly transform into death-promoting organelles, which accelerates the progression of renal fibrosis (Galluzzi et al. 2012). Recent years, NR4A1 has been found to play a critical role in the maintenance of energy homeostasis (Safe et al. 2016). Various overexpression and knockdown studies have shown a key role for NR4A1 in mitochondrial function. For example, NR4A1-overexpressing transgenic muscle mice showed a significant increase in oxidative metabolism and mitochondrial activity (Chao et al. 2012). Overexpression of NR4A1 in C2C12 myoblasts increased mitochondrial content and activity, whereas knockdown of NR4A1 in C2C12 cells and primary muscle cells significantly decreased uncoupling protein 3 expression (Maxwell et al. 2005). Knockdown of NR4A1 reduced high-fat diet-mediated mitochondrial dysfunction in the non-alcoholic fatty liver, including reduced mitochondrial potential, oxidative stress, mitochondrial respiratory depletion, and ATP deficiency (Zhou et al. 2018). These studies clearly indicate that NR4A1 can directly affect mitochondrial activity. However, little is known regarding the effect of NR4A1 on mitochondrial function in the kidney.

Thus, we examined the function of mitochondria in the kidney of UUO rats, whose main function is to support the TCA cycle and aerobic respiration through OXPHOS and to produce ATP through mitochondrial ETC to meet the energy required for cell survival (Wallace 1999; Newmeyer and Ferguson-Miller 2003). CS is a key enzyme and the first rate-limiting enzyme in the mitochondrial TCA cycle, and decreased CS expression directly affects the process of the TCA cycle, leading to decreased ATP production, excessive superoxide production, and apoptosis (Cai et al. 2017). SDH is the only enzyme complex involved in both the TCA cycle and ETC, and a decreasing the activity of SDH leads to an increased formation of ROS (Rasheed and Tarjan 2018). SDHB gene encodes one of the four subunits of succinate dehydrogenase and succinate dehydrogenase activity is usually assessed qualitatively by immunohistochemical staining of SDHB protein (Rasheed and Tarjan 2018). We found that abnormal expression of these key metabolic enzymes in UUO affects the TCA cycle, ultimately leading to decreased ATP production and more ROS synthesis, which are involved in the development of CKD (Figure 4). YHTR and eplerenone could significantly increase these key metabolic enzymes in 14-day UUO rats (Figure 4). In HK2 cells, YHTR pre-incubation could upregulate CS and SDHB expression inhibited by TGF-β1 *via* promoting NR4A1 expression (Figure 6).

Reduced expression of enzyme complex subunits of the mitochondrial ETC have been found in renal tissue (Shoubridge 2001). In physiological conditions, the mitochondrial ETC consists of five multi-enzyme complexes distributed across the mitochondrial cristae, including complex I (NADH dehydrogenase complex), complex II (SDH complex), complex III (cytochrome reductase complex), complex IV (cytochrome oxidase complex), and complex V (ATP synthase complex). ETC complexes I–IV utilizes the reduction potential of NADH and FADH2 generated during the TCA cycle to pump protons into the mitochondrial membrane gap, thereby generating an electrochemical gradient that is then used by complex V to assist in the phosphorylation of ADP to ATP (Mandavilli et al. 2002). While in this process, electron deletion on the ETC can allow partial conversion of oxygen to ROS, an important second messenger that at low levels has a physiological function as a key signalling molecule; however, at higher levels, mitochondrial ROS can be pathogenic, leading to cellular damage (Galvan et al. 2017). Excess ROS induces oxidative stress and promotes pathophysiological processes, such as apoptosis, inflammation, and fibrosis, which are directly related to the initiation and progression process of CKD (Duni et al. 2019). Gene defects in OXPHOS-related genes causing mitochondrial energy metabolism disorders are detected in each component of the ETC complex. It is proposed that the function of each complex can affect the output of the whole OXPHOS process (Mayr et al. 2015). Thus, we analyzed the activity of the complex (I–V) in the ETC of UUO rats. We found that the activity of the mitochondrial complex (I–V) was remarkably reduced in those UUO rats compared to sham group rats and increased by YHTR and eplerenone (Figure 3).

Moreover, excessive production of ROS would damage mtRNA, leading to impaired ETC function, reduced ATP synthesis, and increased mitochondrial outer membrane permeability and cytochrome C release, which would activate caspase-9 with the participation of apoptotic factors, such as Bax and Bid and anti-apoptotic factor Bcl-2, and caspase-9 would be further cleaved to caspase-3, which eventually induced apoptosis (Li et al. 2019). We further analyzed the expression of apoptosis-related proteins and observed a marked decrease in the protein expression of anti-apoptotic factor Bcl-2 and a significant increase in the protein expression of caspase-9 and caspase-3 in kidney of rats 14 days after UUO compared with in sham group (Figure 5). Our data demonstrated that mitochondrial dysfunction leads to apoptosis and there is compelling evidence that apoptosis is involved in the progression of tubular atrophy, renal fibrosis, and CKD (Jadot et al. 2017). It was shown that in pancreatic β-cells, the continuous high glucose environment causes excessive mitochondrial production of ROS and induces oxidative stress, and NR4A1 is resistant to apoptosis triggered by oxidative stress through signalling pathways such as p-JNK, WT1, Survivin and GPX-1 (Mazuy et al. 2013; Zong et al. 2017; Yang et al. 2018). Mitochondrial function and expression of key mitochondrial proteins are altered in accordance with abnormal NR4A1 of expression (Maxwell et al. 2005; Zhou et al. 2018). Furthermore, we speculated that the function of renal mitochondria could be improved by regulating NR4A1 expression. *In vitro*, YHTR could promote NR4A1 expression in HK2 cells, which improved the function of renal mitochondria by increasing expression of CS and SDHB, thus leading to inhibition of TGF-β1-induced cell apoptosis (Figure 6). YHTR significantly ameliorated the development and progression of CKD.

## Conclusion

This study demonstrates the therapeutic effect of YHTR on renal function injured by UUO. YHTR treatment can improve renal mitochondrial function by increasing the activity of the mitochondrial complex (I–V) and key metabolic enzymes, which were regulated by increased NR4A1 expression, and then leading to inhibition of apoptosis in kidneys of UUO rats. Thus, YHTR is a promising drug for the potential treatment of CKD.

## Data Availability

The data used to support the findings of this study are available from the corresponding author upon request.
